# Curcumin Nanoemulsion: Unveiling Cardioprotective Effects via ACE Inhibition and Antioxidant Properties in Hypertensive Rats

**DOI:** 10.3390/medicina59101748

**Published:** 2023-09-29

**Authors:** Mohd Ishaq, Mohemmed Faraz Khan, Garima Verma, Akshoo Rathi, Mohammad Adil, Mohammad Faizan, Abul Kalam Najmi, Mohd Akhtar, Omkulthom Al kamaly, Samar Zuhair Alshawwa, Abdelaaty A. Shahat, Abdulsalam Alhalmi

**Affiliations:** 1 Department of Pharmacology, School of Pharmaceutical Education and Research, Jamia Hamdard, New Delhi 110062, India; mohd.ishaq010@gmail.com (M.I.); rathiakshoo@gmail.com (A.R.); adil4pharma@gmail.com (M.A.); mohd.faijan1314@gmail.com (M.F.); aknajmi@jamiahamdard.ac.in (A.K.N.); 2 Department of Pharmaceutical Chemistry, Faculty of Pharmacy, Integral University, Lucknow 226026, India; faraz91khan@gmail.com; 3 Department of Pharmaceutical Chemistry, School of Pharmaceutical Education and Research, Jamia Hamdrad, New Delhi 110062, India; garimahansrajverma@gmail.com; 4 Department of Pharmaceutical Sciences, College of Pharmacy, Princess Nourah bint Abdulrahman University, P.O. Box 84428, Riyadh 11671, Saudi Arabia; omalkmali@pnu.edu.sa (O.A.k.); szalshawwa@pnu.edu.sa (S.Z.A.); 5 Department of Pharmacognosy, College of Pharmacy King Saud University, P.O. Box 2457, Riyadh 11451, Saudi Arabia; ashahat@ksu.edu.sa; 6 Department of Pharmaceutics, School of Pharmaceutical Education and Research, Jamia Hamdard, New Delhi 110062, India; asalamahmed5@gmail.com

**Keywords:** curcumin, renin angiotensin aldosterone system, angiotensin-converting enzyme, deoxycorticosterone acetate, uninephrectomy

## Abstract

*Background and Objectives*: Curcumin, derived from *Curcuma longa*, is a well-known traditional medicinal compound recognized for its therapeutic attributes. Nevertheless, its efficacy is hampered by limited bioavailability, prompting researchers to explore the application of nanoemulsion as a potential alternative. *Materials and Methods*: This study delves into the antihypertensive effects of curcumin nanoemulsion (SNEC) by targeting the renin-angiotensin-aldosterone system (RAAS) and oxidative stress in deoxycorticosterone acetate (DOCA) salt-induced hypertensive rats. To gauge the cardio-protective impact of SNEC in DOCA salt-induced hypertension, molecular docking was undertaken, uncovering curcumin’s high affinity and adept binding capabilities to the active site of angiotensin-converting enzyme (ACE). Additionally, the investigation employed uninephrectomized rats to assess hemodynamic parameters via an AD instrument. Serum ACE, angiotensin II, blood urea nitrogen (BUN), and creatinine levels were quantified using ELISA kits, while antioxidant parameters were evaluated through chemical assays. *Result*: The outcomes of the molecular docking analysis revealed robust binding of curcumin to the ACE active site. Furthermore, oral administration of SNEC significantly mitigated systolic, diastolic, and mean arterial blood pressure in contrast to the DOCA-induced hypertensive group. SNEC administration also led to a reduction in left ventricular end-diastolic pressure (LVEDP) and an elevation in the maximum rate of left ventricular pressure rise (LV (dP/dt) max). Moreover, SNEC administration distinctly lowered serum levels of ACE and angiotensin II compared to the hypertensive DOCA group. Renal markers, including serum creatinine and BUN, displayed a shift toward normalized levels with SNEC treatment. Additionally, SNEC showcased potent antioxidant characteristics by elevating reduced glutathione, catalase, and superoxide dismutase levels, while decreasing the concentration of thiobarbituric acid reactive substances. *Conclusions*: Collectively, these findings underscore that curcumin nanoemulsion exerts noteworthy cardio-protective effects through ACE activity inhibition and remarkable antioxidant properties.

## 1. Introduction

Cardiovascular diseases stand as a leading cause of mortality on a global scale. The Global Burden of Disease Study 2017 reported that approximately 17.8 million individuals lost their lives due to cardiovascular conditions, accounting for 31% of all global deaths [1]. Among these, hypertension, often referred to as the “silent killer”, is a prevalent concern. It is typically characterized by sustained elevations in systolic blood pressure (>140 mmHg) and diastolic blood pressure (>90 mmHg). Major risk factors contributing to hypertension include dyslipidemia, smoking, excessive alcohol consumption, stress, insulin resistance, and the presence of metabolic syndrome [2].

Renin, a fundamental component of the Renin Angiotensin Aldosterone System (RAAS), originates from the prohormone pro-renin. Its primary role involves catalyzing the conversion of the precursor protein angiotensinogen into angiotensin I. Initially, angiotensin I is an inactive decapeptide, but it undergoes hydrolysis in the presence of angiotensin-converting enzyme (ACE) to transform into the active octapeptide angiotensin II. The ACE inhibition peptide is usually used for hypertension control to prevent the conversion of angiotensin I to angiotensin II [3]. Angiotensin II serves as a potent vasoconstrictor, primarily interacting with the AT-1 receptor. Additionally, Angiotensin II stimulates the adrenal cortex, prompting the release of aldosterone. This hormone targets the principal cells of the collecting ducts in the nephron, enhancing the reabsorption of both salt and water. This process results in an increase in blood volume, leading to elevated blood pressure [4].

Metabolic syndrome is a cluster of interconnected risk factors, including obesity, high blood pressure, elevated blood sugar levels, and abnormal lipid profiles. These factors contribute to an increased risk of cardiovascular disease, type 2 diabetes, and other health problems. Oxidative stress is believed to play a role in the development and progression of metabolic syndrome [5]. The excessive production of free radicals can lead to inflammation, insulin resistance, and damage to blood vessels, all of which are key components of metabolic syndrome. Peroxynitrite (ONOO−), nitrogen oxide (NO*), and hypochlorous acid (HOCl) are produced in everyday metabolic pathways and scavenged through antioxidants [6]. Superoxide dismutase (SOD) and glutathione peroxidase levels are decreased in newly identified and untreated hypertensive patients, leading to higher oxidative stress. Hydrogen peroxide production is also greater in hypertensive people [7,8]. Understanding the link between oxidative stress and metabolic syndrome is essential for developing effective preventive and therapeutic strategies to manage this complex health condition.

Curcumin, a polyphenol found in the rhizome of the herb *Curcuma longa* (turmeric), has various pharmacological activities, including cardioprotective, anti-inflammatory, antioxidant, antiseptic, and antimalarial activities. It also helps with pain management [9,10]. Curcumin may activate various enzymes involved in the detoxification of electrophilic merchandise of lipid peroxidation (LPO), including glutathione-S-transferase (GST) and glutathione peroxidase (GPx) in rats. This property can also be attributed to its antioxidant activities, which is useful for its antihypertensive recreation [11]. Simultaneously, it can also inhibit angiotensin-converting enzyme (ACE) [12]. Thus, drugs with antioxidant properties and ACE inhibitory effects are favored as antihypertensive medicines [13].

Curcumin effectively exhibits its antioxidant capabilities by scavenging free radicals such as O_2_*^−^ and hydroxyl radicals (OH^*^), and by influencing the activity of antioxidant enzymes like GSH, catalase, and SOD, which work to neutralize these radicals [14]. These effects also contribute to its potential in preventing hypertension. One of the key ways through which curcumin exerts its antihypertensive effects is by inhibiting ACE [15].

However, curcumin’s clinical utility is significantly hindered due to its limited bioavailability. Studies have shown that a substantial portion (40–85%) of orally administered curcumin remains unchanged as it passes through the gastrointestinal tract. This poor bioavailability can be attributed to factors like low water solubility, inadequate absorption, and rapid elimination [16]. Researchers have employed various methods to enhance the bioavailability of curcumin. Two common approaches have been pursued to address this challenge. The first approach involves improving curcumin’s transport mechanism (e.g., creating nanoemulsions, curcumin–phospholipid complexes, liposomal curcumin, and chelating with metals). The second approach focuses on altering the structure of curcumin [17].

Based on the current state-of-the-art curcumin formulations and their impact on the pharmacokinetic profile, curcumin formulations have evolved rapidly to improve their pharmacokinetic (PK) profiles. Researchers have explored various innovative approaches to enhance curcumin’s bioavailability and effectiveness. Some state-of-the-art curcumin formulations and delivery systems that have been developed include nanoparticles. Nanoparticle-based curcumin formulations have gained significant attention. Nanoparticles can protect curcumin from degradation, improve its solubility, and enhance its absorption in the body. Lipid-based nanoparticles, polymeric nanoparticles, and solid lipid nanoparticles are examples of such delivery systems [18,19]. Liposomal formulations encapsulate curcumin within lipid bilayers. Liposomes can improve curcumin’s stability and bioavailability by facilitating its transportation across cell membranes [20]. Micelles: Curcumin can also be incorporated into micelles, which are formed by amphiphilic molecules. Micellar formulations enhance curcumin’s solubility and absorption in the gastrointestinal tract [21]. Nanoemulsions: Nanoemulsions are colloidal dispersions (10–100 nm in diameter) of oil and water stabilized by surfactants. They can enhance curcumin’s solubility and stability, leading to improved absorption and bioavailability [22,23]. Phytosomes: Curcumin phytosomes are complexes formed by combining curcumin with phospholipids. This enhances curcumin’s absorption and bioavailability [24]. Curcumin-loaded Microspheres: Microspheres are spherical drug carriers that can release curcumin slowly, leading to prolonged therapeutic effects [25]. Cell-derived vesicles (exosomes): While this technology is relatively new, researchers have been exploring the use of exosomes or cell-derived vesicles in delivering curcumin. These natural vesicles have the potential to improve curcumin’s stability, bioavailability, and targeted delivery [26]. Polymeric Nanocarriers: Various polymeric nanocarriers, including poly(lactic-co-glycolic acid) (PLGA) nanoparticles, have been employed to encapsulate and deliver curcumin and show improved bioavailability [27].

The utilization of a self-nanoemulsifying drug delivery system (SNEDDS) holds the potential to enhance bioavailability and improve the effectiveness of lipophilic drugs like curcumin in treating hypertension [28]. SNEDDS is composed of a mixture of oil, surfactant, and co-surfactant, which, when gently mixed with water, forms a fine oil-in-water (O/W) nanoemulsion [29]. When taken orally, SNEDDS utilizes the natural motion of the stomach to trigger self-emulsification through agitation [30]. Several researchers have created formulations of self-nanoemulsifying curcumin (SNEC) and shown that its bioavailability is approximately twice as high as that of curcumin in an aqueous form [31,32]. The primary objective of this study was to assess the potential antihypertensive effects of SNEC, which was acquired from Arbro Pharmaceuticals Pvt. Ltd. in the form of SNEC-30 capsules. These effects were then compared with those of established standard drugs: captopril and pure curcumin in an aqueous solution. The hypothesis was that the nanoemulsion formulation of curcumin demonstrates promising attributes as a prospective alternative therapeutic intervention for hypertension.

## 2. Materials and Methods

### 2.1. Docking Studies

All computations were performed on an Intel^®^ Core™ i3-3217U CPU @ 1.80 GHz, (Lenovo, Bangalore, India) capacity processor with a memory of 4 GB RAM running on Windows 8.1 operating system. Glide 5.9, Phase 3.5, and LigPrep 2.6 modules implemented in the Maestro 9.4 (Graphical user interface (GUI)) of Schrodinger’s computational chemistry software were used. Glide 5.9 is designed to help with the docking of potential ligands based on their binding modes and likeness to a given receptor molecule. Interactions between the ligands and the protein residues at the active sites of human angiotensin-converting enzymes were visualized using a ligand interaction diagram in Schrodinger suite version 9.4. The entire docking study was performed following the procedure adopted by Verma et al. [33].

#### 2.1.1. Protein Preparation and Grid Generation

The X-ray crystal structure of the human angiotensin-converting enzyme, along with its complex with a specific inhibitor called Lisinopril, was obtained from the RCSB Protein Data Bank (PDB) under the identifier 1O86. This structure, resolved at a resolution of 2.00 Å, was employed as the foundation for computational investigations. The protein preparation wizard in the Maestro 9.4 software was used to process the protein. During this process, bond orders were assigned, hydrogen atoms were incorporated, formal charges were managed, and water molecules were removed. The hydrogen bonding network was optimized using the exhaustive sampling option. This was followed by protein minimization, limiting the Root Mean Square Deviation (RMSD) from the initial structure to 0.3 Å. The Impact 5.9 software was used for this step, employing the OPLS_2005 force field. The refined protein structure was used to create scoring grids for subsequent docking calculations using the Glide software. The docking grids were generated using default parameters within Glide. The co-crystallized ligand (Lisinopril) served as the basis for determining the center of the grid box, which had dimensions of 16 × 16 × 16 Å. The generation process adhered to default settings and no constraints were introduced during the grid generation process [34].

#### 2.1.2. Ligand Preparation

The three-dimensional coordinates of all the ligands or structures were generated using the Maestro 9.4 graphical user interface (GUI) in Schrodinger. The LigPrep 2.6 tool, coupled with Epik 2.4, was used to process the ligands. This process encompassed expanding protonation and tautomeric states at pH 7.0 ± 2.0. Following this, energy minimization was conducted using the OPLS 2005 force field.

#### 2.1.3. Molecular Docking Studies

Molecular docking investigations of the synthesized compounds were executed using the Glide SP module coupled with Epik state penalties to account for various ionization and tautomeric states. Multiple ligand docking poses were generated, providing a range of potential binding orientations. These diverse poses were thoroughly analyzed to derive meaningful insights and interpretations for the outcomes of the study.

### 2.2. Pharmacodynamic Studies

#### 2.2.1. Materials

Curcumin was obtained from Sigma Aldrich (Steinheim, Germany) while SNEC-30 capsules were purchased from Arbro Pharmaceuticals Pvt. Ltd. Deoxycorticosterone acetate (DOCA) and Captopril were purchased from TCI Chemical Pvt Ltd. Rat Angiotensin II Elisa kit was purchased from Genxbio health services Pvt. ltd. Serum creatinine kit was purchased from ARKRAY Health Pvt. Ltd. Blood urea nitrogen estimation kit was purchased from Beacon Diagnostic Pvt. Ltd. All other chemicals and reagents were of analytical grade and were used without further purification.

#### 2.2.2. Experimental Animals and Research Protocol Approval

This study was carried out on Wistar rats (*Rattus norvegicus*) weighing 200–250 g procured from Jamia Hamdard, India. All animals were kept in a polypropylene cage and maintained on a standard rat chow diet (NIN) and water provided ad libitum in a climate-controlled, light-regulated space with 12-h light and dark cycles in the central animal house facility at Jamia Hamdard. The experimental protocol (Proposal No. 1387) was approved by the Institutional Animal Ethics Committee (IAEC) of Jamia Hamdard, New Delhi, India (Registration No. 173/GO/Re/s/2000/CPCSEA). Research was carried out per the guidelines of the committee for the purpose of control and supervision of experiments on animals (CPCSEA).

#### 2.2.3. Uninephrectomy Methodology

Right kidney removal showed considerable importance in a deoxycorticosterone acetate (DOCA) salt-induced hypertension model. First, the animals were anesthetized via intraperitoneal administration of ketamine (75 mg/kg body weight) and xylazine (20 mg/kg body weight). The skin above the right kidney was shaved and cleaned with betadine. A small incision was made above the midscapular region. The right kidney was identified and the adrenal gland and tissue connected to the kidney were eliminated by tearing the attachment. The kidney was gently pulled out. The renal artery and ureter were tied using a silk thread. Muscles and skin layers were sutured using sterile absorbable sutures.

#### 2.2.4. Experimental Induction of Hypertension

DOCA salt administration in uninephrectomised rats is a well-known model of hypertension. After one week of uninephrectomy (recovery period) in this model, we administered DOCA (25 mg/kg body weight) s.c. twice a week for 4 weeks. 1% NaCl and 0.2% KCl were also given with drinking water. Sodium reabsorption by the kidney increased with DOCA administration, leading to hypovolemia. Vasopressin secretion also increased, causing water retention and vasoconstriction [35].

#### 2.2.5. Experimental Design

The animals were randomly divided into ten groups, with seven animals in each group. The right kidneys of the animals in all the groups, except the control and per se group, were surgically removed. One week after surgery, the rats received subcutaneous injections of DOCA (20 mg/kg) twice a week for 28 days, and their drinking water was supplemented with 1.0% NaCl and 0.2% KCl. The groups were divided as follows:

Group I (Control normal): Rats were given normal saline (1 mL/ kg body weight) orally (p.o.).
Group II (Control toxic): The right kidney was surgically removed but the animals did not receive any pathogenic drug. The rats were treated with normal saline (1 mL/kg).
Group III (Pathogenic): DOCA salt (20 mg/kg) was administered subcutaneously to uninephrectomised animals.
Group IV: Curcumin (60 mg/kg) was orally administered to the pathogenic treated animals.
Group V: Curcumin (90 mg/kg) was orally administered to the pathogenic treated animals.
Group VI: Nanocurcumin (60 mg/kg) was orally administered to the pathogenic treated animals.
Group VII: Nanocurcumin (90 mg/kg) was orally administered to the pathogenic treated animals.
Group VIII: Captopril (30 mg/kg) was orally administered to the pathogenic treated animals.
Group IX: Curcumin (90 mg/kg) was orally per se administered to the control group.
Group X: Nanocurcumin (90 mg/kg) was orally per se administered to the control group.

Test and standard drugs were administered every day from the 15th day to the 28th day of the experiment. At the end of the experiment, hemodynamic parameters were assessed using an invasive method. After measuring hemodynamic parameters, blood samples were collected from the animals through the retro-orbital plexus. The heart and kidneys were extracted. Biochemical parameters of the serum and tissues, as well as histological changes in the heart and kidneys, were observed.

#### 2.2.6. Cannulation of the Carotid and Femoral Arteries

On the last day of the experiment, animals were anesthetized via intraperitoneal injection of ketamine (75 mg/kg body weight) and xylazine (20 mg/kg BW). A small incision (1–2 cm) was made in the neck of the rat for cannulation of the carotid. After identifying the carotid artery, it was gently pulled out, its cephalic end was tied, and the cardiac end was clamped. A Millar Mikro-Tip transducer catheter was inserted into the carotid artery. For cannulation of the femoral artery, a small incision was made in the dermis of the proper thigh. The muscle fiber was cleaned cautiously and the bunch of nerve fiber, femoral artery, and femoral vein were identified. The femoral artery was removed from this bunch. The upper part of the artery towards the paw was tied with a cannula pre-filled with heparinized normal saline inserted into the femoral artery towards the heart [36].

#### 2.2.7. Measurement of Hemodynamic Changes

After cannulation, various hemodynamic parameters, including systolic blood pressure (SBP), diastolic blood pressure (DBP), mean arterial pressure (MAP), heart rate, and left ventricular pressure and left ventricular contractility assessment (dP/dt) were recorded. Femoral artery cannulation was used for assessing arterial blood pressure and heart rate with a pressure transducer (Statham p23Db, Los Angeles, CA, USA) using a Powerlab Data Acquisition System (4/25, AD Instrument, Bella Vista, Australia). The carotid artery was used for measuring cardiac left ventricular function. Millar Mikro-Tip transducer catheter was inserted into the right carotid artery up to the left ventricle to record left ventricular pressure (LVP). The maximum and minimum rates of developed left ventricular pressure (LV (dP/dt) max and LV (dP/dt) min) and left ventricular end-diastolic pressure signals were obtained from primary signals (left ventricular systolic pressure and blood pressure) using a data acquisition system (LabChart 7.3; AD Instrument Pvt. Ltd.) [37,38].

#### 2.2.8. Estimation of Heart and Kidney Weights and Relative Organ Weights

The heart and kidneys of the rat were removed and washed with normal saline. The weights of the heart and kidneys were recorded separately. The relative organ weights of both organs were calculated with respect to body weight.

#### 2.2.9. Biochemical Estimation

After recording the hemodynamic parameters, the animals were sacrificed for biochemical estimation. Serum and a small amount of homogenized cardiac tissue sample from each animal were stored in tubes and used for the biochemical tests.

##### Estimation of Serum Angiotensin-Converting Enzyme (ACE)

This assay is primarily based on the hydrolysis of the substrate hippuryl histidine leucine (HHL) using ACE and measuring the quantity of hippuric acid. Approximately 100 µL of serum sample was added to 150 µL HHL (5 mM). A total of 300 mM NaCl was added to adjust the pH to 8.3. Test and control tubes were incubated, with continuous shaking, at 37 ℃ for 30 min. 0.25 mL of 1 N HCl was added to terminate the reaction. 0.4 mL of pyridine and 0.2 mL of benzene sulfonyl chloride were blended with the aid of an inversion for 1 min and cooled on ice. The yellow color that developed was measured at 410 nm [39,40].

##### Estimation of Serum Angiotensin II

Quantitative determination of serum Angiotensin II (Ang II) was performed using an immunoassay kit purchased from Genxbio Health Services Pvt. Ltd. The concentration of Ang II in 40 µL of each sample was determined according to the manufacturer’s protocol and the sample values were derived from the standard curve. Serum Ang II concentrations were measured in pg/mL.

##### Estimation of Serum Blood Urea Nitrogen and Serum Creatinine

Serum blood urea nitrogen was measured according to the manufacturer’s instructions using reagent kits (Beacon Diagnostic Pvt. Ltd., 424, New Gidc, Kabilpore, Navsari- 396 424. India). Serum creatinine was assayed according to the kit manufacturer’s instructions.

#### 2.2.10. Oxidative Stress Parameters in Cardiac Tissue

##### Estimation of Thiobarbituric Acid Reactive Substances (TBARS)

Levels of thiobarbituric acid reactive substances (TBARS) were measured as malondialdehyde (MDA) according to the method by Ohkawa*,* et al. [41] to determine lipid peroxidation in heart tissue. A 100-µL homogeneous sample was mixed with 0.2 mL of 8.1% sodium dodecyl sulfate, 1.5 mL of 20% acetic acid, and 1.5 mL of 0.8% thiobarbituric acid. This mixture was boiled at 95 ℃ for 60 min. After cooling, 5 mL of *n*-butanol and pyridine (5:1) were added and a pink color complex was formed. The absorbance of the organic layer was measured at 532 nm (Specord 200, Germany), plotted against a standard graph, and expressed in nmol/g tissue.

##### Glutathione (GSH) Estimation

Heart tissue (300–600 mg in weight) was homogenized with 8 mL of 0.02 M EDTA; 1 mL of 50% trichloroacetic acid and 4 mL of double distilled water were then mixed with 5 mL of the homogenate. After proper shaking, the tubes were centrifuged for 15 min at 3000 rpm. After this, 2 mL of supernatant was mixed with 0.1 mL of 0.01 M 5, 5′- dithiobis-(2-nitrobenzoic acid) (DTNB) and 4 mL of 0.4 M Tris buffer (pH 8.9). Absorbance was measured at 410 nm within 5 min of adding the DTNB and compared with a blank reagent containing no homogenate. The amount of glutathione was calculated from the standard curve and expressed in U/mg protein [42].

##### Superoxide Dismutase (SOD) Estimation

Heart tissue (20 mg) was homogenized in 2 mL of potassium phosphate buffer. The homogenate was centrifuged for 20 min at 10,000 rpm and 4 ℃ in a cooling centrifuge. A total of 100 µL of the supernatant was mixed with 1.0 mL of 7.5 mM pyrogallol and 2.85 mL of buffer. The change in absorbance was recorded against a buffer at 420 nm at 1-min intervals for 3 min. One unit of SOD activity was defined as the amount of enzyme required to produce 50% inhibition of pyrogallol auto-oxidation under the assay conditions and expressed in Unit/mg protein [43].

##### Catalase (CAT) Estimation

Catalase activity in cardiac tissue was determined following the method by Claiborne, A., 1985 [44]. Heart tissue was homogenized with 50 mM phosphate buffer (pH 7.4) at a ratio of 1:10. The homogenate was centrifuged at 10000 rpm and 4 ℃ for 20 min; 50 µL of the supernatant was taken and mixed with 2.95 mL of a 19 mM/L solution of H_2_O_2_ in tubes. The decrease in absorbance at 240 nm following the addition of H_2_O_2_ was monitored at 1-min intervals for 3 min. Catalase activity was expressed as nmoles of H_2_O_2_ consumed/min/mg protein.

#### 2.2.11. Histopathological Studies

The kidney and heart tissues obtained from all the experimental groups were washed immediately with 0.9% saline and then fixed in 10% buffered neutral formalin. After fixation, the tissues (kidney and heart) were processed by embedding in paraffin wax. Then, the tissues were sectioned (5–6 μm) and stained with hematoxylin and eosin (H&E). The sections were examined under a light microscope and photomicrographs were taken. All histopathological changes were examined by a pathologist [45].

### 2.3. Statistical Analysis

Statistical analysis was carried out using Graphpad Prism 5.0 (Graphpad software; San Diago, CA, USA). All results were expressed as Mean ± S.E.M. All data were compared using analysis of variance followed by Tukey’s multiple comparison method. Values were considered statistically significant where *p* < 0.01.

## 3. Results

### 3.1. Docking Results

**Figure 1:**
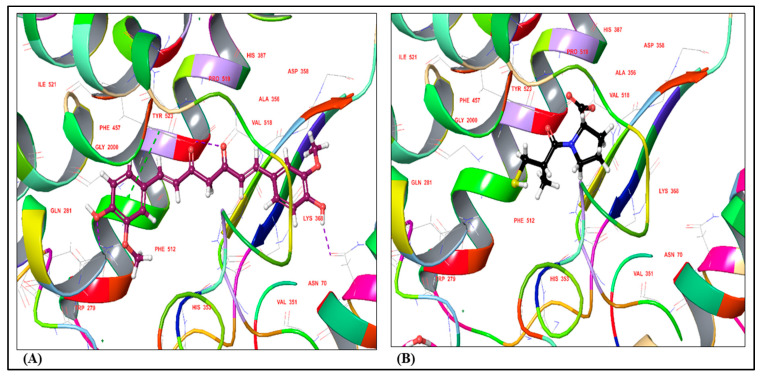
Dock pose for (**A**) Curcumin and (**B**) Captopril with the human angiotensin-converting enzyme (PDB ID: 1O86).

**Figure 2:**
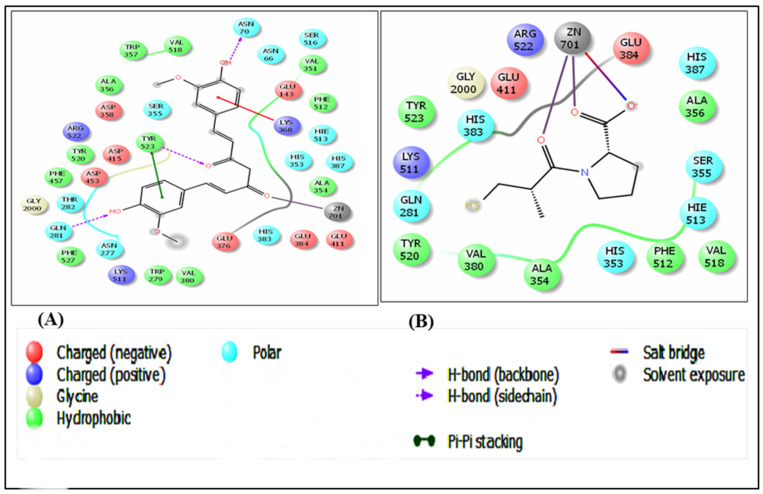
Ligand interaction diagrams (LIDs) of (**A**) Curcumin and (**B**) Captopril with human angiotensin-converting enzyme (PDB ID: 1O86).

Docking studies of all the synthesized compounds were performed using the X-ray crystal structure of the human angiotensin-converting enzyme in complex with a selective inhibitor, Lisinopril (PDB ID: 1O86, Resolution: 2.00 Ǻ) using the GLIDE docking algorithm. Almost all the designed compounds were found to possess noteworthy and remarkable interactions with the protein backbone. Docking results of the compounds indicated that Curcumin exhibited significant molecular interactions compared with the standard drug, Captopril. The dock poses of Curcumin (Dock Score: −6.876) and Captopril (Standard, Dock Score: −7.199) with the protein are shown in Figure 1A and Figure 1B, respectively. Ligand interaction diagrams (LIDs) depicting the molecular interactions between Curcumin and Captopril and the human angiotensin-converting enzyme protein are given in Figure 2A and Figure 2B, respectively. To understand the molecular interactions displayed in LIDs (Figure 2A,B) more clearly, legends for the same are represented in Figure 2C.

### 3.2. Pharmacodynamic Studies

#### 3.2.1. Effect on Body Weight

At the end of the study, the mean weight of rats in the toxic control group (Group II) showed a significant increase compared with the control group. The test groups treated with curcumin (60 mg/kg and 90 mg/kg), nanocurcumin (60 mg/kg and 90 mg/kg), and the standard treatment of captopril (30 mg/kg) showed significant decreases in weight compared with the toxic group.

#### 3.2.2. Effect on Relative Organ Weight

As a quantitative measure of cardiac hypertrophy, we determined the heart weight (mg) to body weight (g) ratios for all the experimental animals. The relative heart weight (heart weight (mg)/body weight (g)) increased significantly in hypertensive animals compared with controls. Treatment with the test drug inhibited the increase in the weight (hypertrophy) of the heart. Table 1 shows the heart–weight ratios. A significant increase in left-kidney-to-body-weight ratio was observed in DOCA control rats compared with the control group. Curcumin administration (60 mg/kg/day and 90 mg/kg/day) attenuated the kidney weight to body weight induced by DOCA salt and significantly decreased the left kidney weight/100 g body weight ratio. Nanocurcumin also reduced the weight of the kidneys.

#### 3.2.3. Effect on Systolic Blood Pressure, Diastolic Blood Pressure, and Mean Arterial Pressure

At the end of the experiment, the pathogenic group (i.e., uninephrectomized rats induced with DOCA salt) showed a significant (*p* < 0.001) increase in SBP, DBP, and MAP compared with the normal control group. Treating the pathogenic group with nanocurcumin (60 mg/kg and 90 mg/kg BW) significantly (*p* < 0.001) prevented an increase in SBP, DBP, and MAP. Curcumin (60 and 90 mg/kg BW) also prevented an increase in SBP, DBP, and MAP compared with the pathogenic group, although its effect was lower than that of nanocurcumin (Table 2).

#### 3.2.4. Effect on Heart Rate

Rats in the pathogenic group (i.e., uninephrectomized rats induced with DOCA salt) showed a significant (*p* < 0.001) increase in HR compared with rats in the control group. Curcumin (60 and 90 mg/kg) significantly (*p* < 0.001) prevented an increase in the HR compared with pathogenic rats. Nanocurcumin 60 mg prevented an increase in heart rate compared with the toxic group, although its effect was much less compared with the nanocurcumin (90 mg/kg) group (Table 2).

#### 3.2.5. Effect on Left Ventricular Function (LVF)

Subcutaneous administration of DOCA salt to uninephrectomised rat showed a significant increase (*p* < 0.05) in left ventricular end-diastolic pressure (LVEDP) and Tau, but significant decreases (*p* < 0.05) in Max dp/dt, Min dp/dt, and contractility index compared with normal rats. Treatment with nanocurcumin (60 and 90 mg/kg) significantly attenuated (*p* < 0.05) the elevated LVEDP and Tau, but significantly increased (*p* < 0.05) Max dp/dt, Min dp/dt, and contractility index compared with toxic control rats (Table 3).

#### 3.2.6. Effect on Serum ACE Levels

Table 4 shows the effects of curcumin and curcumin nanoemulsion on serum levels of angiotensin-converting enzyme in control and DOCA salt-treated uninephrectomised rats. It was observed that serum ACE levels increased in DOCA salt-treated hypertensive rats compared with control rats. Oral administration of curcumin (60 and 90 mg/kg) significantly (*p* < 0.001) reduced ACE levels in DOCA salt-treated hypertensive rats. Curcumin nanoemulsion had a significantly (*p* < 0.001) higher ACE-reducing effect compared with curcumin (Table 4).

#### 3.2.7. Effect on Serum Ang II Levels

Table 5 shows the effect of curcumin and its nanoemulsion on the levels of angiotensin II in the sera of control and DOCA salt-treated uninephrectomised hypertensive rats. Elevated levels of Ang II were observed in hypertensive rats compared with those of control rats. Oral administration of curcumin (60 and 90 mg/kg) significantly (*p* < 0.001) reduced the levels of Ang II in DOCA salt-treated hypertensive rats. Curcumin nanoemulsion showed significantly (*p* < 0.001) higher Ang II-reducing effect compared with curcumin.

#### 3.2.8. Effect on Serum Renal Function Marker

Table 6 summarizes the effects of curcumin nanoemulsion on the levels of renal function markers such as blood urea nitrogen and creatinine in the sera of control and DOCA salt-induced hypertensive rats. The activities of these pathophysiological markers were significantly elevated in DOCA salt-induced hypertensive rats. Treatment with nanocurcumin (60 and 90 mg/kg) significantly (*p* < 0.001) reduced the levels of these markers towards normal. The comparison between curcumin and nanocurcumin was not significant. The data are indicated as mean ± SEM.

#### 3.2.9. Effect on Oxidative Stress Parameters in Cardiac Tissues

**Figure 3:**
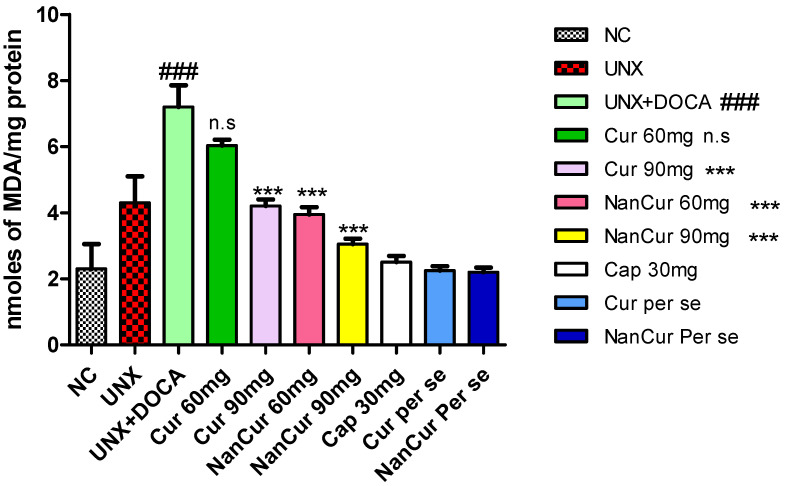
Effect of curcumin nanoemulsion on TBARS in DOCA salt-induced renal hypertensive rats. The TBARS data were analyzed using one-way ANOVA followed by Tukey’s multiple comparison method. The results for each group are given as mean ± SEM (*n* = 7) and markers show significant differences at *p* < 0.05. ^###^ *p* < 0.001 toxic group compared with the control group. *** *p* < 0.01 nanocurcumin (60 mg) compared with the toxic group. *** *p* < 0.001 nanocurcumin (90 mg) compared with the toxic group and n.s. represent non-significant curcumin 60 mg compared with pathogenic group.

**Figure 4:**
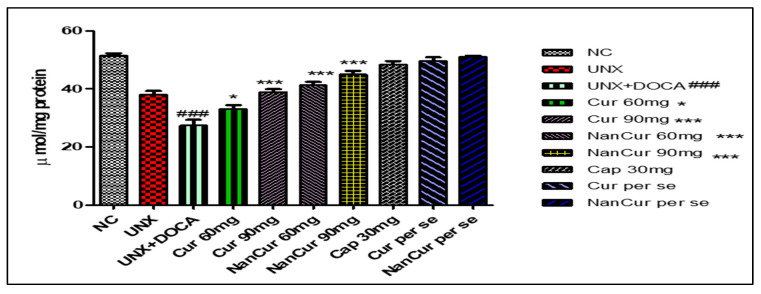
Effect of curcumin nanoemulsion on GSH in DOCA salt-induced renal hypertensive rats. The GSH data were analyzed using one-way ANOVA followed by Tukey’s multiple comparison method. The results for each group are given as mean ± SEM (*n* = 7) and markers show significant differences at *p* < 0.05. ^###^ *p* < 0.001 toxic group compared with the control group. *** *p* < 0.01 nanocurcumin (60 mg) compared with the toxic group. *** *p* < 0.001 nanocurcumin (90 mg) compared with the toxic group, and * curcumin 60 mg is compared with pathogenic group with few change.

**Figure 5:**
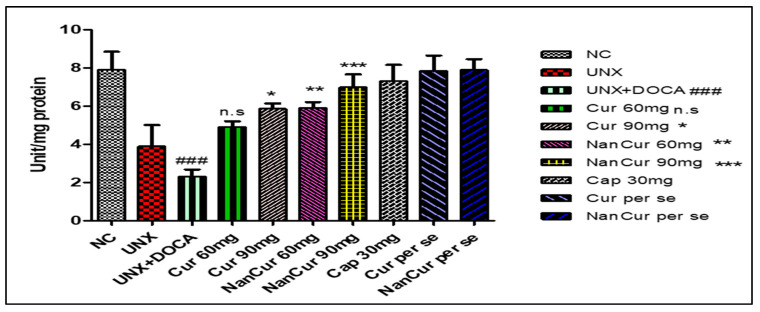
Effect of curcumin nanoemulsion on SOD in DOCA salt-induced renal hypertensive rat. The SOD data were analyzed using one-way ANOVA followed by Tukey’s multiple comparison method. The results for each group are given as mean ± SEM (*n* = 7) and markers show significant differences at *p* < 0.05. ^###^ *p* < 0.001 toxic group compared with the control group. ** *p* < 0.01 nanocurcumin (60 mg) compared with the toxic group. ***p < 0.001 nanocurcumin (90 mg) compared with the toxic group, * means when curcumin 90 mg administered it shows few changes when compared with pathogenic group, and n.s. represent non-significant curcumin 60 mg compared with pathogenic group.

**Figure 6:**
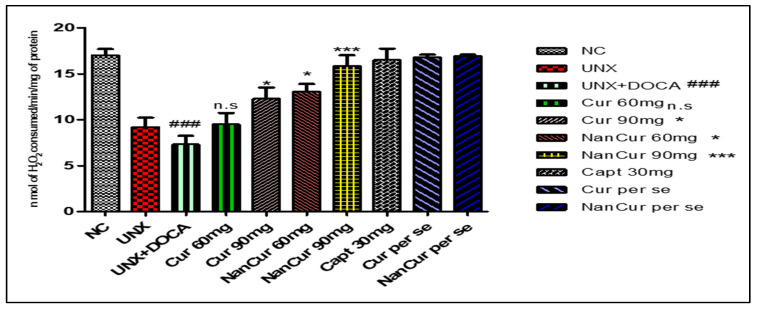
Effect of curcumin nanoemulsion on catalase in DOCA salt-induced renal hypertensive rats. The catalase data were analyzed using one-way ANOVA followed by Tukey’s multiple comparison method. The results for each group are given as mean ± SEM (*n* = 7) and markers show significant differences at *p* < 0.05. ^###^ *p* < 0.001 toxic group compared with the control group. *** *p* < 0.01 nanocurcumin (60 mg) compared with the toxic group. *** *p* < 0.001 nanocurcumin (90 mg) compared with the toxic group, * means when curcumin 90 mg administered it shows few changes when compared with pathogenic group, and n.s. represent non-significant curcumin 60 mg compared with pathogenic group.

Cardiac MDA levels were significantly (*p* < 0.001) elevated in pathogenic rats. Simultaneously, the activities of SOD, GSH, and catalase decreased significantly (*p* < 0.001) compared with normal control rats. Treatment with nanocurcumin impeded the variations in antioxidant enzymes and thus reduced oxidative stress. Administration of curcumin (60 mg) did not show any significant changes in the levels of cardiac lipid peroxide, SOD, and catalase compared with pathogenic rats. However, this curcumin dose significantly (*p* < 0.05) increased the level of GSH. Curcumin (90 mg) significantly (*p* < 0.001) inhibited variations in the levels of MDA, GSH, SOD, and catalase (*p* < 0.05) (Figure 3, Figure 4, Figure 5 and Figure 6).

### 3.3. Histopathological Examination

**Figure 7:**
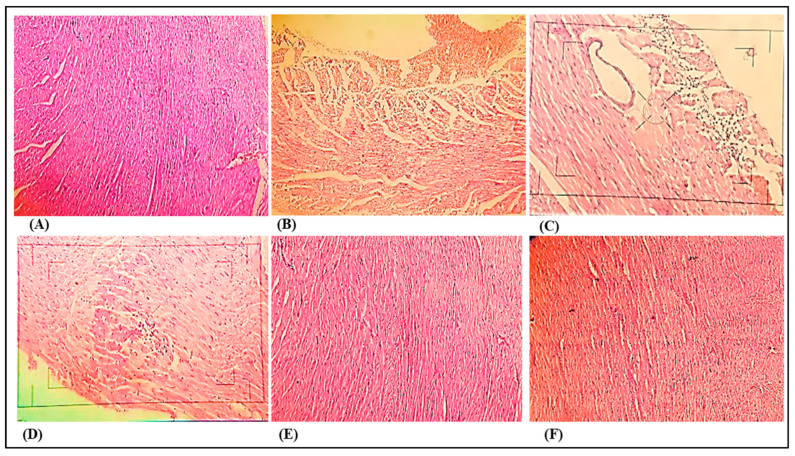
Photomicrographs of heart sections. (**A**) Photomicrograph of control group rat’s heart section, showing normal myocardial heart (10×). (**B**) Photomicrograph of DOCA salt-induced pathogenic group rat’s heart section, showing loss of muscle fibers (10×). (**C**) Photomicrograph of DOCA salt-induced pathogenic group rat’s heart section, showing inflammatory and fatty cells (40×). (**D**) Photomicrograph of nanocurcumin (60 mg/kg) rat’s heart section, showing some inflammatory cells and loss of muscle fibers (10×). (**E**) Photomicrograph of nanocurcumin (90 mg/kg) rat’s heart section, showing slight myocardial degeneration and a small degree of inflammatory cell processes (10×). (**F**) Photomicrograph of nanocurcumin (per se) rat’s heart section, showing normal cardiac muscle fibers in the subendothelial zone (10×).

Figure 7 illustrates the histological transformations observed in the hearts of both control rats and rats with DOCA salt-induced hypertension. Histopathological analysis of heart tissues taken from rats subjected to DOCA induction and uninephrectomy displayed extensive changes in myocardial structure, along with subendocardial necrosis accompanied by interstitial edema and infiltration of leukocytes. In contrast, the control group exhibited no such structural modifications. Notably, therapeutic administration of nanocurcumin at a dosage of 90 mg/kg demonstrated a significant enhancement in cases of DOCA salt-induced uninephrectomized rats, evident by reduced subendocardial necrosis, capillary dilatation, and leukocyte infiltration. Treatment with nanocurcumin at a dosage of 60 mg/kg exhibited minor myocardial degeneration and a slight degree of inflammatory cell activity.

**Figure 8:**
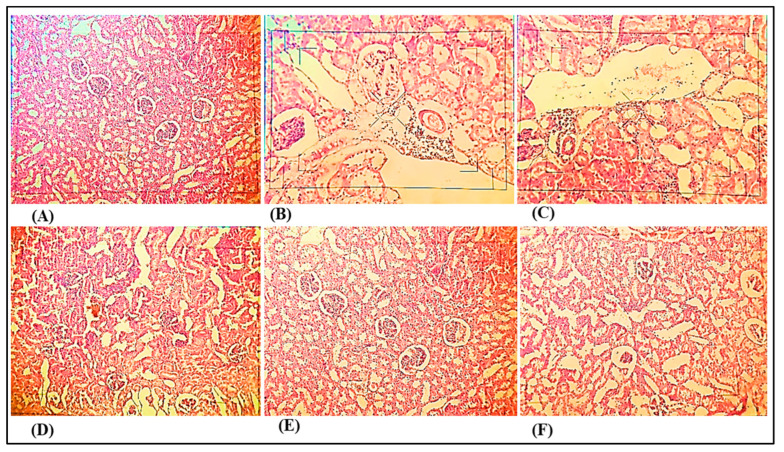
Photomicrographs of kidney sections. (**A**) Photomicrograph of control group rat’s kidney section showing normal glomeruli, tubules, and tubular epithelial (40×). (**B**) Photomicrograph of DOCA salt-induced pathogenic group rat’s kidney section showing atrophy of tubular cells and congestion of glomerulus (40×). (**C**) Photomicrograph of DOCA salt-induced pathogenic group rat’s kidney section showing dilated lumen of distal convoluted tubules (40×). (**D**) Photomicrograph of nanocurcumin (60 mg/kg) rat’s kidney section showing reduced congestion of glomerulus and focal tubular necrosis. (40×). (**E**) Photomicrograph of nanocurcumin (90 mg/kg) rat’s kidney section showing minimal congestion of glomerulus and focal tubular necrosis (40×). (**F**) Photomicrograph of nanocurcumin (per se) rat’s kidney section showing normal glomeruli, tubules, and tubular epithelial (40×).

Figure 8 shows histological changes in the kidneys of control and DOCA salt-induced hypertensive rats. Histopathological examination of kidney tissues from DOCA salt-induced uninephrectomised rats showed atrophy of tubular cells with dilated lumen of distal convoluted tubules and congestion of glomerulus in comparison with the control group where no such structural alterations were observed. Treatment with nanocurcumin (60 mg/kg) demonstrated reduced congestion of the glomerulus and focal tubular necrosis. Treatment with nanocurcumin (90 mg/kg) showed minimal congestion of the glomerulus and focal tubular necrosis.

## 4. Discussion

To gain deeper insights into the interaction between curcumin and the active site residue of angiotensin-converting enzyme, a molecular docking study was conducted. The docking analyses for all the synthesized compounds were executed using the X-ray crystal structure of human angiotensin-converting enzyme complexed with the selective inhibitor, lisinopril (PDB ID: 1O86, Resolution: 2.00 Å). The GLIDE docking algorithm was employed for these studies. Notably, nearly all the designed compounds exhibited significant and remarkable interactions with the protein backbone.

Curcumin is a phenolic compound that can treat or prevent various diseases such as Alzheimer’s, multiple sclerosis, cataract formation, rheumatoid arthritis, fibrosis, and pulmonary toxicity [46,47]. Curcumin has a number of beneficial effects on the cardiovascular system, including blood pressure lowering effect in L-NAME-induced hypertensive rats [48]. However, curcumin has poor biopharmaceutical properties such as low solubility, poor permeability, and extensive first-pass metabolism [49]. These obstacles are major hurdles in its clinical application because they reduce its bioavailability. However, this limitation has been overcome by formulating SNED of curcumin. SNED is a novel technique for improving the bioavailability of hydrophobic drugs such as curcumin. It has been reported that the bioavailability of SNEC is much better than that of curcumin [50].

In this study, we induced hypertension using uninephrectomy and a DOCA salt hypertensive model. This model is widely used for evaluating the cardioprotective effect of various drugs [51,52].

The hemodynamic parameters were assessed using an AD instrument. Systolic blood pressure, diastolic blood pressure, mean arterial pressure, and heart rate were observed by cannulating the femoral artery. We observed that these hemodynamic readings were increased in the pathogenic group while curcumin shifted these readings toward normal. Our study corroborated previous findings by Berthon et al. [53]. We also observed that nanoemulsion of curcumin (60 and 90 mg/kg) significantly decreased systolic B.P, diastolic B.P, mean arterial pressure, and heart rate compared with pathogenic rats. This indicates that curcumin nanoemulsions (SNEC) have antihypertensive action against DOCA-induced hypertension. It was also observed that the antihypertensive effect of nanocurcumin was better than that of commercially obtained curcumin.

Left ventricular function was observed by cannulating the carotid artery. DOCA administration significantly increased the LVEDP and decreased LV (dP/dt) max. A rise in LVEDP showed an increased preload or incomplete emptying of the left ventricles, thus left ventricle performance was impaired [54]. When ventricular performance is impaired, cardiac output is depressed, indicating that there is a deterioration in cardiac performance, which can be a sign of heart failure [55]. Administration of nanocurcumin (60 and 90 mg) significantly increased the LV (dP/dt) max and reduced the LVEDP, which showed that blood flow was restored to the subendocardial region of the heart and reduced towards normal via reduction of elevated LVEDP.

The kidney plays a pivotal role in regulating the balance between body salt and water. An altered salt–water balance causes disordered regulation of renal functions, which may lead to hypertension [56]. Hypertensive rats elevate plasma/serum levels of urea, uric acid, and creatinine, which are considered significant markers of renal function. Renal marker levels were significantly disturbed in the sera of DOCA-induced hypertensive rats; after treatment with nanocurcumin, these marker levels shifted significantly toward normal levels.

Oxidative stress plays a crucial role in both the commencement and progression of cardiovascular dysfunction. Elevated levels of reactive oxygen species (ROS) such as superoxide anion, hydrogen peroxide, and lipid peroxides become prevalent during instances of oxidative stress, thereby fostering the development of hypertension. In this context, the balance between ROS production and the efficacy of antioxidant defense mechanisms becomes perturbed, thereby magnifying the influence of oxidative stress [57]. The generated ROS induces oxidative deterioration in polyunsaturated fatty acids (PUFAs), which are linked with altered membrane structure and enzyme inactivation. Thus, lipid peroxidation is an important pathogenic event during hypertension. Thiobarbituric acid reactive substances (TBARS) and lipid hydroperoxides are the end products of lipid peroxidation; their concentrations were highly elevated in the plasma and tissues of DOCA-induced rats, indicating high oxidative stress in DOCA-induced rats. Nanocurcumin (60 and 90 mg/kg) treatment decreases the levels of lipid peroxidation products in DOCA-induced rats. This evidence suggests that the antihypertensive action of nanocurcumin would be at least partially due to its antioxidant potential.

The activities of SOD, CAT, and GSH were significantly decreased in rats induced with DOCA compared with rats in the control group. This decline indicates a reduced capacity to counteract active oxygen species and reactive metabolites, implying a weakened defense mechanism against oxidative stress in DOCA-induced hypertensive rats.

Histopathological analysis of hearts treated with DOCA revealed extensive modifications in the myocardial structure. These changes encompassed cardiac myocyte hypertrophy, separation of cardiac muscle fibers, interstitial edema, fibrosis, necrosis, and infiltration of inflammatory cells, consistent with findings from prior research. On the other hand, hearts treated solely with curcumin per se and nanocurcumin per se displayed normal cardiac fibers without any pathological indications, suggesting that neither curcumin nor nanocurcumin had a notable impact on the myocardium itself. However, when nanocurcumin was administered orally as a therapeutic intervention, it notably ameliorated the cardiac injury induced by DOCA, thus confirming its beneficial effects in protecting the heart.

Histopathological examination of kidneys in rats induced with DOCA salt reveals several notable findings, including tubular cell atrophy, dilation of distal convoluted tubule lumens, and glomerular congestion. In contrast, animals treated with nanocurcumin exhibit normal glomerular and tubular epithelial morphology. This observation confirms the nephroprotective effect of nanocurcumin and its potential in hypertension treatment.

## 5. Conclusions

The present study evaluated the antihypertensive effect of curcumin nanoemulsion in DOCA salt-induced hypertension and compared its efficacy with curcumin and captopril, a standard drug. The DOCA salt-induced model served as an important model for renal hypertension profiling as shown by alterations in the blood pressure. Curcumin decreased blood pressure—as shown by the hemodynamic parameters—and reduced Ang II, suggesting that curcumin may be a new adjuvant and/or agent that may have potential in treating hypertension. Nanoemulsion of curcumin showed greater antihypertensive responses compared with curcumin based on hemodynamic parameters and reduced Ang II levels in the serum. Oxidative stress was observed in DOCA salt-treated animals and was able to reduce the levels of GSH and CAT, and the activity of SOD in the cardiac tissue alone and when drinking water was supplied with 1% NaCl and 0.5% KCl. Curcumin and nanocurcumin increased the levels of GSH, CAT, and SOD. The DOCA salt-treated group showed high TBARS values compared with the normal group. Curcumin decreased TBARS levels significantly. Nanocurcumin showed better antioxidant activity compared with curcumin. Serum creatinine and serum blood urea nitrogen levels were increased in the DOCA-treated group compared with normal controls. Nanocurcumin reduced the levels of these renal markers to normal. Histopathological examinations also showed improvement in the nanocurcumin-treated rats, indicating cardioprotective effects. Thus, based on the above finding, it can be concluded that curcumin nanoemulsion showed better antihypertensive activity, improved KFT, reduced oxidative stress parameters, and improved histopathological alterations compared with curcumin per se. Thus, it may be a potential adjuvant due to its cardioprotective and antihypertensive activities. Our results need further evaluation in different animal models of hypertension to provide a conclusive view.

## Figures and Tables

**Table 1:** Effect of curcumin and nanocurcumin on body weight and relative organ weight in DOCA salt-induced uninephrectomised renal hypertensive rats.

S. No.	Groups	Initial Body Weight (g) Ist Day	Final Body Weight (g) after 5 Weeks	Kidney Weight to Body Weight (mg/g) Ratio	Heart Weight to Body Weight (mg/g) Ratio
I	Control	250	290	3.32 ± 0.04	3.29 ± 0.05
II	UNX	230	310	3.31 ± 0.05	3.26 ± 0.03
III	UNX + DOCA	245	283	4.21 ± 0.06 ^###^	4.02 ± 0.06 ^###^
IV	Curcumin 60 mg	260	275	3.76 ± 0.04 ^$$$^	3.81 ± 0.05 ^$$$^
V	Curcumin 90 mg	250	276	3.38 ± 0.02 ^$$$^	3.45 ± 0.05 ^$$$^
VI	Nanocurcumin 60 mg	245	265	3.34 ± 0.02 ***	3.39 ± 0.04 ***
VII	Nanocurcumin 90 mg	240	270	3.26 ± 0.02 ***	3.26 ± 0.04 ***
VIII	Captopril 30 mg	230	25	2.43 ± 0.03	3.16 ± 0.06
IX	Per se Curcumin	250	255	3.20 ± 0.03	3.18 ± 0.02
X	Per se Nanocurcumin	265	270	3.38 ± 0.02	3.34 ± 0.03

Values are expressed as mean ± SEM (*n* = 7). Significant differences set at *p* < 0.001 (ANOVA followed by Tukey’s multiple comparison method. ^###^ *p* < 0.001 toxic group compared with controls. *** *p* < 0.001 Nanocurcumin group compared with DOCA salt-induced toxic group. ^$$$^ *p* < 0.00 Curcumin compared with nanocurcumin.

**Table 2:** Effect of curcumin and nanocurcumin on SBP, DBP, MAP, and heart rate in DOCA salt-induced uninephrectomised renal hypertensive rats.

S. No.	Group	SBP (mmHg)	DBP (mmHg)	MAP (mmHg)	Heart Rate (beats/min)
I	Control	128.56 ± 2.80	84.32 ± 1.42	100 ± 3.98	181 ± 7.25
II	UNX	139.85 ± 2.332	90.12 ± 2.05	108.20 ± 5.60	195.71 ± 3.68
III	DOCA + UNX	193.94 ± 3.52 ^###^	142.11 ± 2.98 ^###^	193.45 ± 5.75 ^###^	281.28 ± 6.00 ^###^
IV	Curcumin 60 mg	145.31 ± 2.36 ^$$$^	90.65 ± 2.41 ^$$$^	118 ± 5.78 ^$$$^	202.42 ± 10.78 ^$$$^
V	Curcumin 90 mg	136.94 ± 2.42 ^$$$^	93.75 ± 3.15 ^$$$^	106.85 ± 3.45 ^$$$^	191.42 ± 7.23 ^$$$^
VI	Nanocurcumin 60 mg	131.49 ± 1.50 ***	84.03 ± 2.88 ***	104 ± 5.04 ***	196.57 ± 5.25 ***
VII	Nanocurcumin 90 mg	125.31 ± 1.50 ***	84.05 ± 2.88 ***	94.85 ± 5.04 ***	170.28 ± 5.25 ***
VIII	Captopril 30 mg (std)	127.92 ± 2.90	88.49 ± 3.93	99.57 ± 6.06	183.71 ± 9.05
IX	Curcumin per se	129.45 ± 2.44	85.14 ± 3.33	95.14 ± 6.28	184 ± 5.64
X	Nanocurcumin per se	125 ± 1.25	86.57 ± 5.88	95.14 ± 5.14	190.57 ± 5.44

Values are expressed as mean ± SEM (*n* = 7). Significant differences set at *p* < 0.001 (ANOVA followed by Tukey’s multiple comparison method. ^###^ *p* < 0.001 toxic group compared with controls. *** *p* < 0.001 Nanocurcumin group compared with DOCA salt-induced toxic group. ^$$$^ *p* < 0.00 Curcumin compared with nanocurcumin.

**Table 3:** Effect of curcumin and nanocurcumin on left ventricular function in DOCA salt-induced uninephrectomised renal hypertensive rats.

S. No.	Groups	LVEDP (mmHg)	Max dp/dt (mmHg/s)	Min dp/dt (mmHg/s)	Contractility Index (1/s)	Tau (ms)
I	Control	7.05 ± 0.80	3950 ± 248.2	−2510 ± 131.7	54.78 ± 2.15	4.89 ± 0.22
II	UNX	8.05 ± 0.85	3200 ± 180.5	−2250 ± 119.75	38.30 ± 2.44	6.55 ± 1.05
III	UNX + DOCA ^###^	12.50 ± 1.45 ^###^	2608 ± 145.0 ^###^	−1810 ± 175.30 ^###^	23.20 ± 1.63 ^###^	10.10 ± 1.45 ^###^
IV	Curcumin 60 mg ^$$$^	11.75 ± 1.06 ^$$$^	3410 ± 195.5 ^$$$^	−1915 ± 181.30 ^$$$^	29.10 ± 1.72 ^$$$^	7.55 ± 0.80 ^$$$^
V	Curcumin 90 mg ^$$$^	10.69 ± 1.10 ^$$$^	3520 ± 248.5 ^$$$^	−2110 ± 190.50 ^$$$^	32.40 ± 1.05 ^$$$^	6.99 ± 0.70 ^$$$^
VI	Nanocurcumin 60 mg ***	10.00 ± 0.75 ***	3484 ± 230 ***	−2280 ± 120.90 ***	35.86 ± 2.05 ***	7.96 ± 0.5 ***
VII	Nanocurcumin 90 mg ***	8.05 ± 0.88 ***	3790 ± 210.06 ***	−2340 ± 152.4 ***	42.85 ± 2.51 ***	5.99 ± 1.22 ***
VIII	Captopril 30 mg	7.55 ± 0.69	3890 ± 230.13	−2300 ± 148.70	43.10 ± 2.81	5.00 ± 0.87
IX	Per se Curcumin	7.30 ± 0.10	3676 ± 252.11	−2455 ± 120	48.50 ± 2.60	5.55 ± 0.95
X	Per se Nanocurcumin	7.66 ± 0.90	3600 ± 261.15	−2411 ± 119.10	47.50 ± 2.19	4.30 ± 0.65

Values are expressed as mean ± SEM (*n* = 7). Significant differences set at *p* < 0.001 (ANOVA followed by Tukey’s multiple comparison method. ^###^ *p* < 0.001 toxic group compared with controls. *** *p* < 0.001 Nanocurcumin group compared with DOCA salt-induced toxic group. ^$$$^ *p* < 0.00 Curcumin compared with nanocurcumin.

**Table 4:** Effect of curcumin and nanocurcumin serum ACE levels in DOCA salt-induced uninephrectomised renal hypertensive rats.

S. NO.	Group	Serum ACE Level (µmole/g.min)
I	Control	2.5 ± 0.20
II	UNX	3.5 ± 0.20
III	UNX + DOCA	6.62 ± 0.35 ^###^
IV	Curcumin 60 mg	4.2 ± 0.22 ^n.s^
V	Curcumin 90 mg	3.9 ± 0.29 **
VI	Nanocurcumin 60 mg	3.9 ± 0.23
VII	Nanocurcumin 90 mg	2.7 ± 0.30 ***
VIII	Captopril 30 mg	2.6 ± 0.19
IX	Per se Curcumin	2.7 ± 0.20
X	Per se Nanocurumin	2.7 ± 0.22

Values are expressed as mean ± SEM (*n* = 7). Significant differences set at *p* < 0.001 (ANOVA followed by Tukey’s multiple comparison method. ^###^ *p* < 0.001 toxic group compared with the control group. *** *p* < 0.001 Nanocurcumin group compared with DOCA salt-induced toxic group. n.s. (non-significant) curcumin 60 mg compared with pathogenic group. ** curcumin 90 mg compared with pathogenic groups.

**Table 5:** Effect of curcumin and nanocurcumin serum Ang II levels in DOCA salt-induced uninephrectomised renal hypertensive rats.

S. No	Group	Serum Ang II Level (pg/mL)
I	Control	60.42 ± 4.64
II	UNX	65.71 ± 3.97
III	UNX + DOCA	79.42 ± 4.94 ^###^
IV	Curcumin 60 mg	75.85 ± 4.18 ^n.s^
V	Curcumin 90 mg	74.85 ± 4.21 **
VI	Nanocurcumin 60 mg	72.28 ± 3.56
VII	Nanocurcumin 90 mg	64.28 ± 3.56 ***
VIII	Captopril 30 mg	62.71 ± 3.51
IX	Per se Curcumin	63.85 ± 5.55
X	Per se Nanocurumin	61.00 ± 5.12

Values are expressed as mean ± SEM (*n* = 7). Significant differences set at *p* < 0.001 (ANOVA followed by Tukey’s multiple comparison method. ^###^ *p* < 0.001 toxic group compared with the control group. *** *p* < 0.001 Nanocurcumin group compared with DOCA salt-induced toxic group. . n.s. (non-significant) curcumin 60 mg compared with pathogenic group, and ** curcumin 90 mg compared with with pathogenic groups.

**Table 6:** Effect of curcumin and nanocurcumin on serum creatinine and blood urea nitrogen in DOCA salt-induced uninephrectomised renal hypertensive rats.

S. No.	Groups	Serum Creatinine (mg/dL)	Blood Urea Nitrogen (mg/dL)
I	Control	1.01 ± 0.19	21.42 ± 3.63
II	UNX	1.41 ± 0.03	27 ± 1.7
III	UNX + DOCA	2.22 ± 0.17 ^###^	59.52 ± 3.08 ^###^
IV	Captopril 60 mg	1.50 ± 0.09 ^n.s^	44 ± 2.77 ^n.s^
V	Captopril 90 mg	1.36 ± 0.11 ^n.s^	42.42 ± 3.30 ^n.s^
VI	Nanocurcumin 60 mg	1.33 ± 0.13 **	39.28 ± 4.28 **
VII	Nanocurcumin 90 mg	1.28 ± 0.13 ***	31 ± 4.28 ***
VIII	Captopril	1.29 ± 0.18	28.85 ± 3.60
IX	Per se Curcumin	1.21 ± 0.21	24.71 ± 2.69
X	Per se Nanocurcumin	1.12 ± 0.12	19 ± 3.13

Values are expressed as mean ± SEM (*n* = 7). Significant differences set at *p* < 0.001 (ANOVA followed by Tukey’s multiple comparison method. ^###^ *p* < 0.001 toxic group compared with the control group. *** *p* < 0.001 Nanocurcumin group compared with DOCA salt-induced toxic group, . n.s. (non-significant) curcumin 60 mg compared with pathogenic group, and ** curcumin 90 mg compared with with pathogenic groups.

## Data Availability

Not applicable.

## Abbreviations

UNX	Uninephrectomised
Cur	Curcumin
Nanocur	Nanocurcumin
SNEC	Self-nanoemulsifying curcumin
SNEDDS	Self-nanoemulsifying drug delivery system
RAAS	Renin–angiotensin–aldosterone system
ACE	Angiotensin-converting enzyme
BUN	Blood urea nitrogen
DOCA	Deoxycorticosterone acetate
LVEDP	Left ventricular end-diastolic pressure
SBP	Systolic blood pressure
DBP	Diastolic blood pressure
MAP	Mean arterial pressure
Cap	Captopril
ROS	Reactive oxygen species
TBARS	Thiobarbituric acid reactive substances
GSH	Glutathione
CAT	Catalase
SOD	Superoxide dismutase
s.c	Sub cutaneous
p.o.	Per oral
i.p	Intraperitoneal
